# Mouse Sphingosine Kinase 1a Is Negatively Regulated through Conventional PKC-Dependent Phosphorylation at S373 Residue

**DOI:** 10.1371/journal.pone.0143695

**Published:** 2015-12-07

**Authors:** Yong-Seok Oh, Sun Sik Bae, Jong Bae Park, Sang Hoon Ha, Sung Ho Ryu, Pann-Ghill Suh

**Affiliations:** 1 Department of Brain-Cognitive Science, Daegu-Gyeongbuk Institute of Science and Technology (DGIST), Hyeonpung-myeon, Dalseong-gun, Daegu, Republic of Korea; 2 Department of Life Science, Division of Molecular and Life Science, Pohang University of Science and Technology, Pohang, Republic of Korea; 3 Department of Biological Sciences, Ulsan National Institute of Science and Technology (UNIST), Ulsan, Republic of Korea; University of Pittsburgh School of Medicine, UNITED STATES

## Abstract

Sphingosine kinase is a lipid kinase that converts sphingosine into sphingosine-1-phosphate, an important signaling molecule with intracellular and extracellular functions. Although diverse extracellular stimuli influence cellular sphingosine kinase activity, the molecular mechanisms underlying its regulation remain to be clarified. In this study, we investigated the phosphorylation-dependent regulation of mouse sphingosine kinase (mSK) isoforms 1 and 2. mSK1a was robustly phosphorylated in response to extracellular stimuli such as phorbol ester, whereas mSK2 exhibited a high basal level of phosphorylation in quiescent cells regardless of agonist stimulation. Interestingly, phorbol ester-induced phosphorylation of mSK1a correlated with suppression of its activity. Chemical inhibition of conventional PKCs (cPKCs) abolished mSK1a phosphorylation, while overexpression of PKCα, a cPKC isoform, potentiated the phosphorylation, in response to phorbol ester. Furthermore, an *in vitro* kinase assay showed that PKCα directly phosphorylated mSK1a. In addition, phosphopeptide mapping analysis determined that the S373 residue of mSK1a was the only site phosphorylated by cPKC. Interestingly, alanine substitution of S373 made mSK1a refractory to the inhibitory effect of phorbol esters, whereas glutamate substitution of the same residue resulted in a significant reduction in mSK1a activity, suggesting the significant role of this phosphorylation event. Taken together, we propose that mSK1a is negatively regulated through cPKC-dependent phosphorylation at S373 residue.

## Introduction

Sphingolipids such as ceramide, sphingosine (SPH), and sphingosine-1-phosphate (S1P) are ubiquitous constituents of eukaryotic membranes that regulate cell growth, survival, apoptosis, differentiation, migration, and immune responses [1–4]. In contrast to ceramide and SPH, which are associated with apoptosis, S1P has been clearly established as a pro-survival molecule [5], as well as an important regulator of cellular trafficking, differentiation, angiogenesis, and inflammation [5]. S1P acts as both an intracellular second messenger and an extracellular ligand [1–4, 6, 7]. Inside cells, S1P is important for direct modulation of the activity of histone deacetylase [7], the ubiquitin ligase activity of TRAF2 [8], activation of MAP kinase [9], and Ca^2+^ mobilization [10, 11]. In another context, S1P functions as an extracellular ligand for a family of S1P-specific cell-surface G protein-coupled receptors (GPCRs) [5, 12]. In addition, S1P is generated in and released from multiple types of cells [1]. Five S1P receptors (S1P_1-5_) interact with S1P at the plasma membrane and then signal downstream via various G proteins including G_q_, G_i/o_, and G_12/13_, allowing for cell type-specific responses [1, 5, 12].

Sphingosine kinase (SK) is a lipid kinase that converts SPH into S1P by ATP-dependent phosphorylation [3]. The level of S1P in the cell is regulated in response to extracellular stimuli, probably by adjusting the balance between SK-mediated synthesis and degradation by SPP lyase or phosphatase [1]. To date, it is not clear that the activity of S1P lyase or phosphatase is transiently regulated; by contrast, many studies have established that the activity of cellular SK is regulated dynamically in the context of cellular physiology [3]. Indeed, SK is activated by multiple stimuli, including as PDGF [13], serum [13, 14], TNFα [15], NGF [16], VEGF [17], acetylcholine [18, 19], phorbol ester [20], forskolin [21], and FcgRII ligation [22], and formyl peptide [23]. On the other hand, SK activity could be negatively regulated in response to extracellular stimuli. For example, HDL profoundly inhibits TNF-stimulated sphingosine kinase activity in endothelial cells, resulting in decreased S1P production [24]. Despite extensive studies about the physiological roles of SK and its product S1P, the molecular mechanisms underlying SK regulation have remained largely unclear.

The mouse genes, *mSK1* and *mSK2*, encode the mSK1 and mSK2 proteins, respectively, each of which has multiple splice variants that differ only at the N-terminus [1, 5]. Just like the human isoforms, mSK1 and mSK2 exhibit different tissue distributions and subcellular location patterns [25]. Genetic knockout of each SK isoform has confirmed that the two isoforms may have distinct physiological functions [26–29], in addition to some functional redundancy [30, 31]. Except for the catalytic domains, the two SK isoforms differ significantly in protein sequence and molecular structure [1, 32, 33]. mSK1 harbors multiple Ca^2+^/calmodulin-binding sequences [32] and both isoforms have a proline-rich motif that might be involved in the interaction with SH3 domain-containing protein(s) [33], suggesting that the two isoforms might be differentially regulated through both specific and common signaling pathways. However, the possibility that each mSK isoform is regulated by distinct phosphorylation event has not been explored yet.

In this study, we analyzed the phosphorylation pattern of mSK isoforms in response to extracellular stimuli. Based on the initial observation that mSK1a is robustly phosphorylated in response to phorbol ester, we characterized the molecular pathway leading to mSK1a phosphorylation and elucidated its influence on SK activity.

## Material and Methods

### Materials

S1P and SPH were from Biomol (Enzo Life Sciences, US). [^32^P]ATP was purchased from PerkinElmer NEN Products (Boston, MA, USA). Phorbol 12-myristate 13-acetate (PMA), 4-deoxy pyridoxine, GF109204X, Go6976, and RO-31-8220 were from Merck Millipore (San Diego, USA). Silica TLC G60 plate was from Merck Millipore. Serum and medium were obtained from HyClone (Logan, UT), and other chemicals were from Sigma-Aldrich (St. Louis, USA).

### Cell culture

COS-7 cells (from ATCC) were maintained in Dulbecco's modified Eagle's medium supplemented with 10% FBS. Cells were grown at 37°C in humidified atmosphere containing 5% CO_2_.

### Immunoblot Analysis

Cell lysates (30 μg) were resolved by 12% SDS-PAGE and transferred to NC membrane (Schleicher & Schuell Bioscience). Membranes were blocked by incubation for 30 min at room temperature with T-TBS (TBS and 0.1% (v/v) Tween 20) containing 5% (w/v) nonfat milk. Primary antibodies were incubated overnight at 4°C in the blocking buffer at the appropriate dilutions. The bound primary IgGs were detected by incubation with secondary antibodies conjugated to HRP and developed using the ECL system (GE Healthcare Life Sciences).

### Plasmid construction and mutagenesis

IMAGE clones 4221357 (*mSK1a*) and 2650442 (*mSK2*) were obtained from the Human Genome Mapping Project Resource Centre in UK and verified by direct sequencing with appropriate primers. Full-length cDNAs were amplified with PCR using primer pairs for mSK1a (5’-gc gaa ttc atg gaa cca gaa tgc cct cga-3’/5’-gc gga tcc tta tgg ttc ttc tgg agg tgg- 3’) and mSK2 (5’-ccc aag ctt atg gcc cca cca cca cta ctg-3’/ 5’-ccg gaa ttc tca ggc ttg tgg ctt ttg acc-3’). The PCR products were digested with *Eco*RI/*Bam*HI (for mSK1a) or *Hin*dIII/*Eco*RI (for mSK2), and subsequently ligated into pCMV2-FLAG.

Site-directed mutagenesis was carried out using the splice-overlap extension method. For the substitution of S373 with alanine, PCR was carried out with wild-type (WT) mSK1a as the template DNA, the forward primer 5'- ggc cgg gac gcc cgg cgg ggg- 3’, and the reverse primer 5’- ccc ccg ccg ggc gtc ccg gcc-3’. From the PCR product, the *Eco*RI/*Bam*HI fragment was cloned into pCMV2-FLAG. The strategy was used to generate the other mutations using the following oligonucleotides: forward primer, 5’-aga ccc gcc gcc aca ctg gtg-3’/reverse primer, 5’-cac cag tgt ggc ggc ggg tct-3’ (S225A); forward primer, 5’-gag ccc agg gcc cag agg ggc-3’/reverse primer, 5’-gcc cct ctg ggc cct ggg ctc-3’ (S332A); forward primer, 5’-ggc cgg gac gcg cgg cgg ggg-3’/reverse primer, 5’-ccc ccg ccg cgc gtc ccg gcc-3’ (S373A); and forward primer, 5’-ggc cgg gac gag cgg cgg ggg-3’/reverse primer, 5’-ccc ccg ccg ctc gtc ccg gcc-3’ (S373E). All mutations were verified by direct sequencing of the entire ORFs while confirming the absence of undesired mutations.

### Transfection

COS-7 cells were plated on 35 mm or 10 cm culture dishes at a density of 1.5 × 10^5^ or 7 × 10^5^ cells/dish, respectively. The next day, 1–4 μg of plasmid DNAs (pCMV2-control, pCMV2-mSK2, pCMV2-mSK1a WT, pCMV2-mSK1a S373A, pCMV2-mSK1a S373E, pCMV2-mSK1a S225A, pcDNA3.1, pcDNA3.1 PKCα, or pcDNA3.1 PKCδ) were transfected using the Lipofectamine 2000^™^ reagent (Life Technologies). Transfected cells were serum-deprived for 24 hrs before agonist stimulation and then stimulated with agonists for the indicated times.

### Measurement of SK Activity

#### *In vitro* SK assay

As described previously with minor modifications [34], the cells were washed with ice-cold PBS and scraped in SK assay buffer (20 mM Tris buffer [pH 7.2], 10 mM MgCl_2_, 20% glycerol, 1 mM dithiothreitol, 1 mM EDTA, 1 mM Na_3_VO_4_, 15 mM NaF, 10 μg/ml leupeptin and aprotinin, 1 mM PMSF, and 0.5 mM 4-deoxypyridoxine). For cell lysis, cells were ruptured by sonication (Branson Sonifier, output control 3) in SK assay buffer supplemented with 0.25% Triton X-100. Cell homogenates were centrifuged at 15,000 rpm to remove the insoluble fraction. SK activity in cell extracts was measured by incubation in SK assay buffer with 50 μM SPH, solubilized in 0.25% Triton X-100 and 1 mM [^32^P] ATP for 20 min at 37°C. The labeled lipids were extracted and resolved by TLC in the solvent of 1-butanol/ethanol/acetic acid/water (8:2:1:2). The formation of S1P was visualized and quantitated on a PhosphoImager system (Fuji Film, Tokyo).

#### Measurement of [^3^H] S1P formation

COS-7 cells, transfected with either control or mSK1a constructs, were serum-deprived for 24 hrs. The transfected cells were equilibrated in DMEM containing 1 mg/ml fatty acid-free BSA for 30 min, and then the medium was replaced with serum-free DMEM containing [^3^H]SPH (5 × 10^5^ cpm, final concentration ~15 nM) preloaded onto 1 mg/ml fatty acid-free BSA, together with PMA (100 nM) or vehicle. After incubation for 5 min, the assay medium was replaced with 1 ml of pre-chilled methanol. The lipids were extracted, dried, and resolved by TLC in 1-butanol:acetic acid:water (3:1:1) as described previously [18]. SPH (Rf 0.67) and S1P (Rf 0.51) spots were visualized by ninhydrin and molybdenum blue staining. Each spot was scraped off, and radioactivity was measured by liquid scintillation counter.

### Immuno-affinity Purification of mSK1a

After transfection with mSK1a, COS-7 cells were lysed and sonicated in lysis buffer A (20 mM Tris, pH 8.0, 1 mM EDTA/EGTA, 0.1 mM DTT) supplemented with 0.25% Triton X-100 and protease inhibitors (1 mM PMSF, 10 μg/ml leupeptin, and 10 μg/ml aprotinin). The lysates were centrifuged at 15,000 rpm for 20 min at 4°C. The supernatants were incubated at 4°C overnight with constant agitation with 50 μl of α-FLAG (M2 clone) Affi-Gel (Sigma-Aldrich, USA). The resin was washed four times with 1 ml of lysis buffer A. Bound protein was eluted with lysis buffer A containing 4 μg/ml 3×FLAG peptide (Sigma-Aldrich).

### Expression and Purification of PKCα

Recombinant baculovirus expressing PKCα was a generous gift from Dr. W. Cho (University of Illinois). PKCα was expressed in baculovirus-infected Sf9 cells and purified as described with some modifications [35]. Monolayers of Sf9 cells (2 × 10^7^ cells/150 mm dish, ten dishes) were infected at a multiplicity of infection of 10. PKCα was purified using Hitrap-Q FPLC, followed by Phenyl-Superose FPLC (GE Healthcare Life Sciences). The purity of PKCα was assessed higher than 90% by SDS-PAGE and Coomassie stain.

### *In vivo* and *in vitro* ^32^P-labeling mSK1a and Purification

For *in vivo* phosphorylation, COS-7 cells in 60 mm dishes were transfected with mSK1a plasmid and metabolically labeled with [^32^P] inorganic phosphate (0.4 mCi) in 2 ml of phosphate-free DMEM for 3 hrs. After PMA (100 nM) treatment for 10 min, cells were washed twice with ice-cold PBS, lysed in 500 μl of lysis buffer (20 mM Tris [pH 7.4], 1 mM EDTA, 1 mM EGTA, 15 mM NaF, 1 mM Na_3_VO_4_, 30 mM sodium pyrophosphate, 10 mM β-glycerophosphate, 1% Triton X-100, 20% glycerol) containing protease inhibitors, and centrifuged at 15000×*g* for 5 min at 4°C. mSK1a was isolated with α-FLAG Affi-Gel, separated with SDS-PAGE, electro-blotted onto NC membrane, and analyzed by autoradiography.

*In vitro* phosphorylation by PKC was carried out under standard kinase assay conditions. Briefly, recombinant mSK1a (50 ng) was incubated with purified PKCα (10 ng) in the presence or absence of 1 μM PMA in a total volume of 50 μl of PKC assay buffer (50 mM MES [pH 6.5], 1.25 mM EDTA, 12.5 mM MgCl_2_) containing 2.5 μCi of [^32^P]-ATP and 10 μM ATP for the times specified at 30°C. Reactions were terminated by adding 12.5 μl of 5×SDS sample buffer and boiling for 5 min. Proteins were separated by SDS-PAGE and electro-blotted onto NC membrane. The phosphorylated protein bands were localized by autoradiography to PhosphoImager or photographic film.

### Phosphopeptide Mapping and Phospho-amino Acid Analysis

*In vivo* immunoprecipitated or *in vitro* ^32^P-labeled mSK1a was resolved by 10% SDS-PAGE and electro-blotted onto NC membrane. Two-dimensional phosphopeptide mapping of phosphorylated mSK1a and phosphoamino acid analysis were carried out on a Hunter thin layer electrophoresis system (CBS Scientific Company, Del Mar, CA, USA) as described previously [36]. Briefly, phosphorylated mSK1a proteins were separated by SDS-PAGE. mSK1a protein was washed and digested with trypsin for 12 hrs at 37°C before the addition of another 2 μg of trypsin; the total incubation time was 24 hrs. The phosphopeptides were separated by two-dimensional chromatography.

Phosphoamino acid analysis was performed essentially as described previously [36]. Phosphopeptides localized by autoradiography were scraped and eluted from the cellulose matrix with 200 μl of pH 1.9 buffer. Acid-hydrolyzed phosphopeptides were separated by two-dimensional electrophoresis using the HTLE 7000 electrophoresis system. Standard amino acids (serine, threonine, and tyrosine) were visualized with ninhydrin, and the phosphoamino acid content of mSK1a was analyzed by autoradiography.

Phosphate-release assays were performed utilizing Edman degradation chemistry. Aliquots of purified phosphopeptides were covalently linked to a Sequelon AA filter using the Sequelon AA reagent kit (Millipore), and phosphate was extracted from each cycle with three 0.5 ml aliquots of 90% methanol/0.015% phosphoric acid as the solvent. Fractions from each cycle were collected and counted in a liquid scintillation counter.

### Two-dimensional Gel Electrophoresis of mSK1a

A ready-to-use immobilized pH gradient strip (GE Healthcare Life Sciences) was rehydrated for 12 hrs in 2D lysis buffer (7 M urea, 2 M thiourea, 4% CHAPS, 2% DTT, protease inhibitors, and phosphatase inhibitors). mSK1a-containing immune complex was solubilized by 2D lysis buffer with gentle vortexing for 1 hr at room temperature. Sample was applied to the rehydrated strips and run on a horizontal MultiPhorII IEF unit. Second-dimension gel electrophoresis was performed as specified by the manufacturer (GE Healthcare Life Sciences). Immunoblotting was performed as described elsewhere.

### Calmodulin-affinity Binding Analysis

Purified mSK1a proteins (0.5 μg) were incubated with 50 μl of Calmodulin (CaM)-Sepharose 4B beads (GE Healthcare Life Sciences), equilibrated with binding buffer (20 mM HEPES [pH 7.4], 200 mM KCl, 0.1% Triton X-100, 20% glycerol) supplemented with the indicated concentrations of free Ca^2+^ ions. The mixtures were incubated for 3 hrs at 4°C with continuous mixing. Unbound proteins were removed by washing three times with binding buffer containing free Ca^2+^ ion. Proteins bound to CaM–Sepharose beads were boiled in 1×Laemmli sample buffer and separated by SDS-PAGE and electro-blotted onto NC membrane. The blot was probed with α-FLAG antibody. Sepharose CL-4B (GE Healthcare Life Sciences) was used as a negative control to monitor the level of nonspecific binding of mSK1a to the beads themselves.

## Results

### PMA-induced phosphorylation of mSK1a

**Fig 1:**
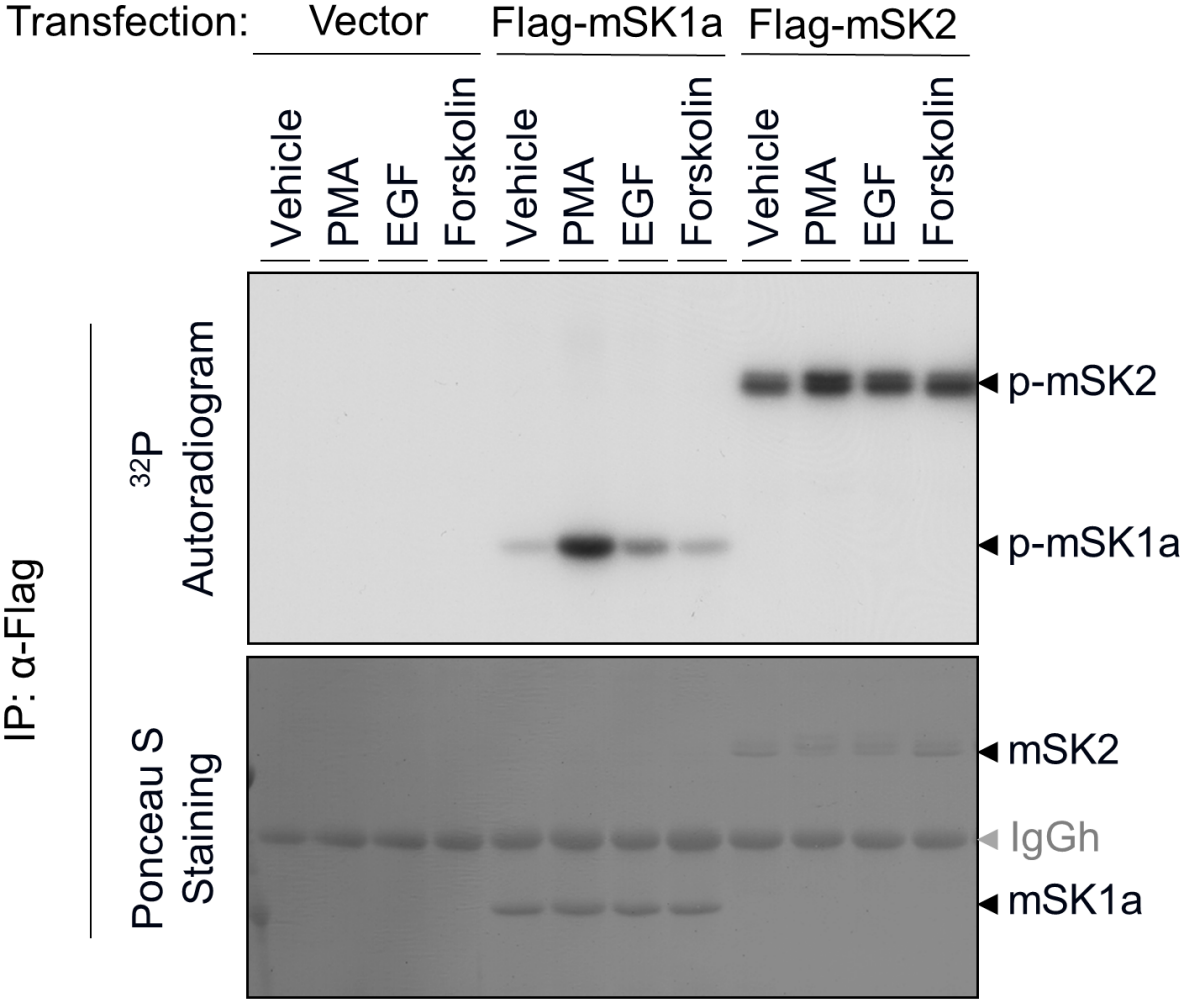
Phosphorylation of mSK isoforms in response to extracellular stimuli. COS-7 cells were transfected with control vector, FLAG-tagged mSK1a, or FLAG-tagged mSK2 constructs. After serum deprivation and metabolic labeling with [^32^P] inorganic phosphate, the cells were treated for 10 min with various agonists, as indicated: PMA (100 nM), EGF (100 ng/ml), or forskolin (20 μM). mSK isoforms were immunoprecipitated using α-FLAG Affi-Gel, transferred on nitrocellulose membrane, and exposed to X-ray photographic film (top panel) after Ponceau S staining (bottom). This result is representative of three independent experiments.

To investigate the mechanism of post-translational regulation of the mouse SK (mSK) isoforms, mSK1a and mSK2, we first examined whether these proteins were phosphorylated in response to external stimuli. After overexpression of either mSK1a or mSK2 and ^32^P metabolic labeling, we treated COS-7 cells with various types of extracellular stimuli, including PMA, forskolin, and epidermal growth factor (EGF), that have been previously identified as activators of human SK isoforms [13, 20, 21, 37, 38]. Consistent with a previous study of hSK isoforms [21, 37, 38], phosphorylation of mSK1a is strongly induced in response to PMA, and to a lesser extent by EGF. By contrast, the basal level of mSK2 phosphorylation remained high, even in quiescent cells subjected to serum withdrawal, and agonist stimulation did not alter mSK2 phosphorylation markedly as shown in mSK1a (Fig 1). These results revealed that the phosphorylation levels of mSK1a and mSK2 are differentially regulated in response to extracellular stimuli. In particular, mSK1a is robustly phosphorylated by PMA stimulation, suggesting a possibility that mSK1a is regulated by this phosphorylation event.

### Inverse correlation between mSK1a phosphorylation and its activity

**Fig 2:**
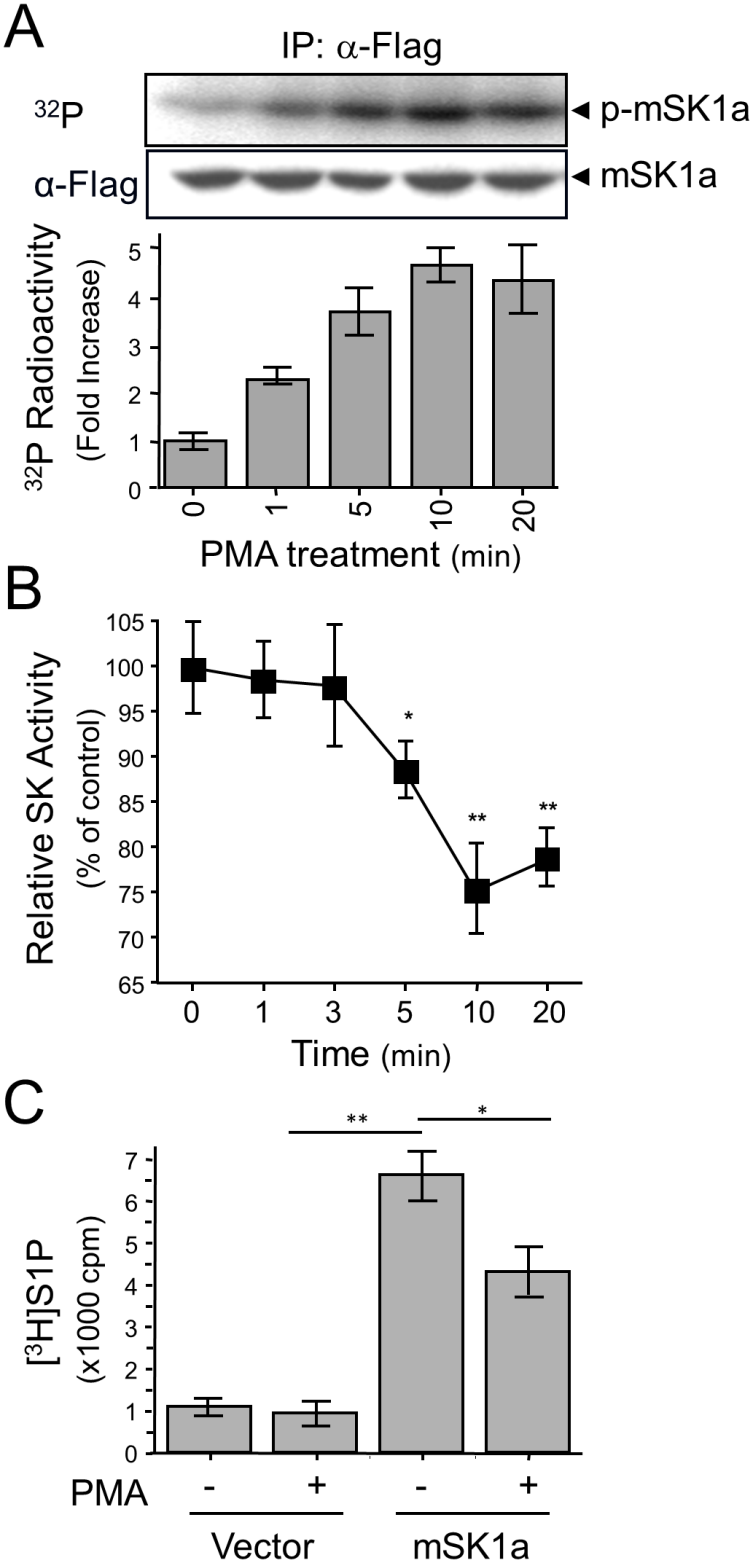
PMA-induced phosphorylation and inhibition of mSK1a. (A) Time-dependent phosphorylation of mSK1a in response to PMA treatment. COS-7 cells were transfected with mSK1a cDNA. After metabolic labeling with [^32^P] *ortho*-phosphate, the cells were treated with 100 nM PMA for indicated periods of time. mSK1a was immunoprecipitated using the FLAG epitope. The autoradiogram and immunoblot images using α-FLAG antibody are shown (top and middle panels), and relative radioactivity was quantitated in duplicate (bottom). Data represent the means ± SE. (B) Time course of PMA-induced suppression of mSK1a activity. COS-7 cells, transfected with mSK1a cDNA, were stimulated with 100 nM PMA for the indicated periods of time. Data are from triplicate determinations from independent cultures, and are expressed as percentages relative to untreated controls. Data represent the means ± SEM. (One-way ANOVA test, *p < 0.05, **p < 0.01). (C) PMA-induced suppression of [^3^H]S1P formation by mSK1a. Formation of [^3^H]S1P was determined in COS-7 cells that are transfected with either control vector or mSK1a cDNA, and further stimulated with 100 nM PMA for 10 min prior to [^3^H]SPH labeling. Data represent the means ± SEM. (t-test, *p < 0.05, **p < 0.01).

Next, we examined the correlation between mSK1a phosphorylation and enzyme activity. When measured over a time course, mSK1a phosphorylation progressively increased in response to PMA treatment up to 4.5-fold (Fig 2A), whereas its *in vitro* activity was suppressed (Fig 2B). Furthermore, we observed that PMA treatment resulted in the marked reduction of cellular S1P formation in the mSK1a-transfected cells, by inhibiting its cellular activity (Fig 2C). These results strongly suggested that mSK1a is negatively regulated by PMA treatment and that phosphorylation of mSK1a is closely related to the suppression of its activity.

### Conventional PKC (cPKC)-dependent phosphorylation of mSK1a

**Fig 3:**
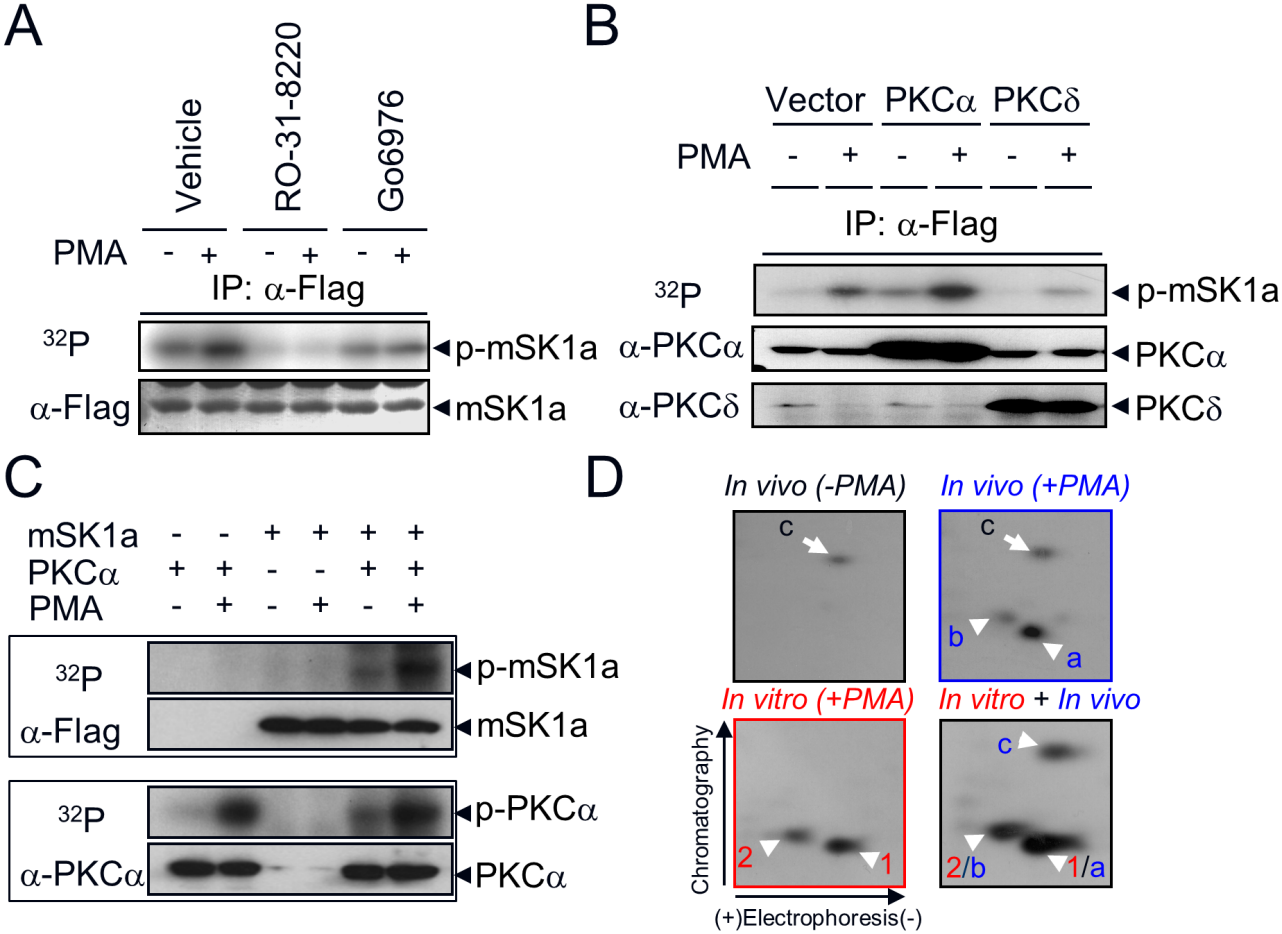
Direct phosphorylation of mSK1a by cPKC. (A) Effect of PKC inhibition on mSK1a phosphorylation. COS-7 cells were transfected with FLAG-tagged mSK1a and then labeled with [^32^P] *ortho*-phosphate. As indicated, the cells were treated with PKC inhibitors (10 μM Ro-31-8220, or 0.5 μM Go6976) for 15 min prior to PMA treatment (100 nM, 10 min). mSK1a immobilized onto nitrocellulose membrane (NC) was exposed to a photographic film (top). The same NC membrane was immunoblotted with anti-FLAG antibody (bottom). (B) Effect of PKC overexpression on PMA-induced mSK1a phosphorylation. mSK1a cDNA were co-transfected with control vector or cDNAs encoding PKCα or PKCδ as indicated. Relative radioactivity of immunoprecipitated mSK1a was visualized as described above (top panel), and the overexpression of each PKC isozyme was confirmed by immunoblot analysis of total homogenates using antibodies specific for each isozyme (PKCα or PKCδ). (C) Direct phosphorylation of mSK1a by purified PKCα. One hundred nanograms of mSK1a and/or 10 ng of PKCα were incubated in 25 μl of phosphorylation buffer containing [^32^P]-ATP in the presence or absence of 1 μM PMA for 20 min as indicated. Incorporation of radioactivity was determined after separation of the reaction mixtures by gel electrophoresis and autoradiography. The upper panel is an autoradiogram of NC membrane corresponding to mSK1a and PKCα, respectively. The lower panel is an immunoblot of the same NC membrane showing that equal amounts of proteins were used in the phosphorylation reactions. (D) Comparison of mSK1a phosphopeptides phosphorylated *in vitro* and *in vivo*. mSK1a phosphorylated *in vivo* was obtained from COS-7 cells after they were treated with PMA (*In vivo* +PMA) or not treated (*In vivo* -PMA). mSK1a (*in vitro* +PMA) phosphorylated by PKCα *in vitro* was digested with TPCK-trypsin. The tryptic digests of *in vivo* and *in vitro* phosphorylated mSK1a, as well as a mixture of the two (*in vivo + in vitro*), were subjected two-dimensional phosphopeptide mapping analysis consisting of electrophoresis and subsequent chromatography. The arrowheads indicate two overlapping phosphopeptides (1/a, 2/b) and the arrow is for non-overlapping peptide (c).

PMA is a potent activator that stimulates multiple subclasses of PKC isoforms, including both conventional (α, βI, βII, γ) and novel (δ, ε, η, θ) isozymes in various cell types [39]. To determine which PKC isozyme mediates PMA-induced mSK1a phosphorylation, we used two independent approaches. First, we examined the effect of pharmacological PKC inhibitors, such as PKC-nonselective RO-31-8220 [40], and PKCα/β1-selective Go6976 [41], on mSK1a phosphorylation. PMA-induced phosphorylation of mSK1a was completely abolished by pretreatment with RO-31-8220 or Go6976 (Fig 3A). Second, we examined the effect of overexpression of each isozyme on mSK1a phosphorylation. mSK1a phosphorylation was significantly increased by overexpression of PKCα, a cPKC isoform, but not PKCδ, a novel PKC isoform (Fig 3B), which suggests the specific PKC pathway mediates mSK1a phosphorylation. These results suggested that the PKCα or possibly other cPKC isozymes mediates PMA-induced phosphorylation of mSK1a in COS-7 cells.

After the functional role of cPKC upstream of mSK1a phosphorylation was established, it remained unclear whether mSK1a is the direct substrate of cPKC. To address this question, we conducted *in vitro* kinase assays by reconstituting purified mSK1a and PKCα in the presence or absence of PMA. mSK1a was directly phosphorylated by PKCα, a cPKC isoform, *in vitro*, as it is *in vivo* (Fig 3C). Furthermore, we compared the 2D-phosphopeptide maps derived from mSK1a proteins phosphorylated *in vitro* and *in vivo* (Fig 3D). PMA treatment of cells overexpressing mSK1a led to the generation of two *in vivo* phosphopeptides (designated a and b). In addition, one basal phosphopeptide (designated as c) was present irrespective of PMA treatment. Notably, *in vitro* phosphorylation of mSK1a generated two phosphopeptides (designated as 1 and 2) that completely overlapped with *in vivo* phosphopeptides a and b, respectively, as revealed by analysis of the mixture (*in vivo* + *in vitro*, designated 1/a and 2/b). By contrast, *in vivo* peptide c, which was not responsive to PMA treatment, was not present in mSK1a phosphorylated by PKCα *in vitro*. These data suggested that PKCα directly phosphorylates mSK1a, at the same residue both *in vivo* and *in vitro*.

### Determination of *in vivo* phosphorylation sites of mSK1a

**Fig 4:**
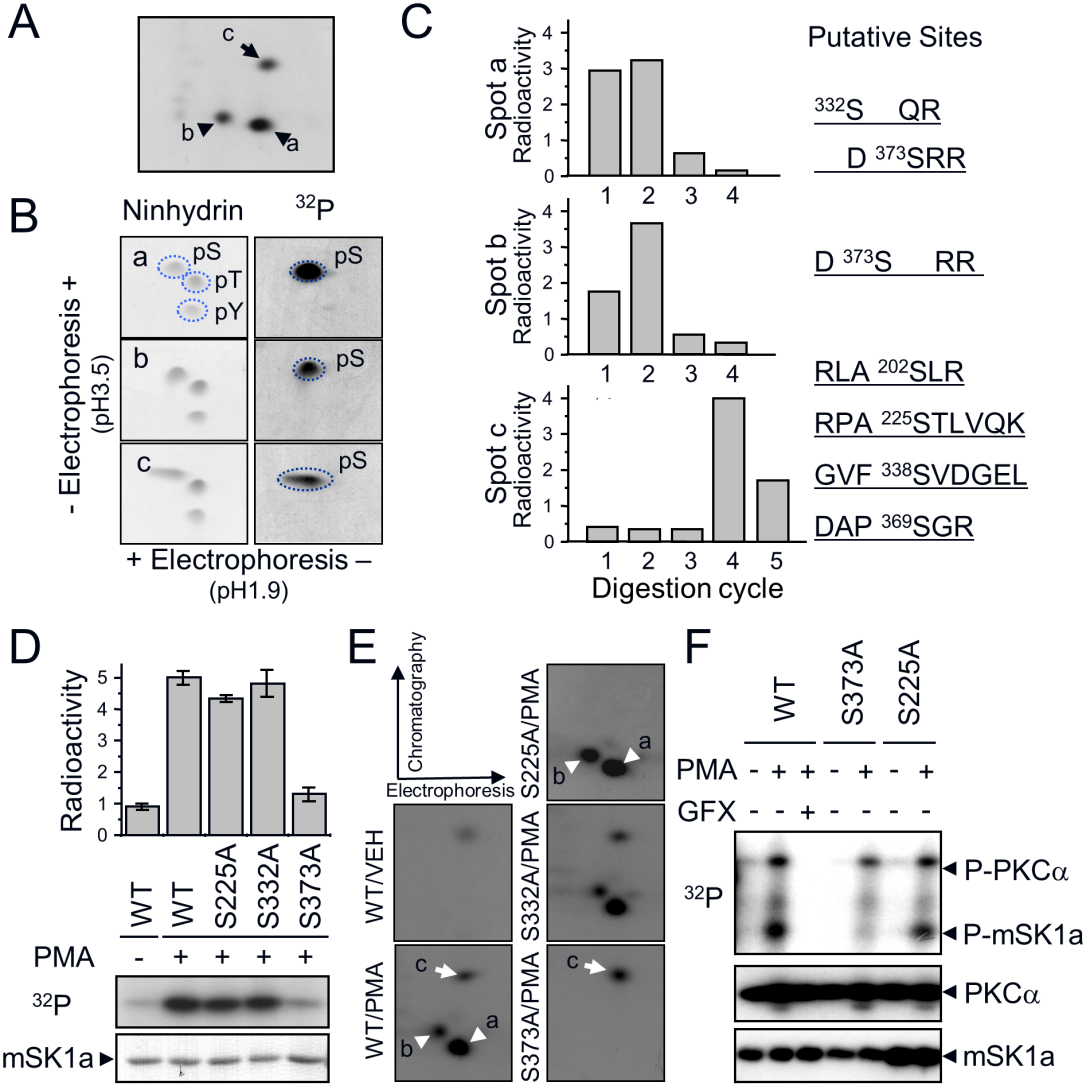
mSK1a S373 is the cPKC-dependent phosphorylation site. (A) PKC-dependent (spots a and b: arrowheads) and -independent (spot c: arrow) phosphopeptide(s) are indicated. Each peptide was eluted from the TLC plate and divided into two fractions, which were subjected to two-dimensional phosphoamino acid analysis or ^32^P-release assay. (B) Phosphoamino acid analysis of three phosphopeptides. Acid-hydrolyzed peptides were subjected to 2-dimensional phosphoamino acid analysis. Dotted circles indicate the migration positions of phosphoserine (pS), phosphothreonine (pT), and phosphotyrosine (pY) standards (left panel) and the image from autoradiography (right panel). (C) ^32^P-release assay of three radiolabeled peptides. Radiolabeled peptides loaded onto the sequencing membrane (Millipore) were digested sequentially from the N-terminus by the Edman degradation method. Putative candidate sites for each phosphopeptide were predicted based on the relative position from the tryptic cleavage site, as shown on the right. (D) Site-directed mutagenesis of candidate sites. COS-7 cells were transfected with FLAG-tagged mSK1a constructs including wild-type (WT) and its mutant (S225A, S332A, S373A) and treated with PMA (100 nM). The relative phosphorylation level of each mSK1a form was assessed in duplicate. Data represent the means ± SE. (E) Two-dimensional phosphopeptide analysis of WT mSK1a and its S/A mutants (S225A, S332A, S373A). cPKC-dependent spots (a and b) and -independent spot (c) are indicated by arrowheads and arrow, respectively. (F) *In vitro* phosphorylation of WT mSK1a and its S/A mutants by PKCα. Wild-type mSK1a and S/A mutants (S373A, S225A) were expressed in COS-7 cells and purified using α-FLAG Affi-Gel. Each protein (100 ng) was incubated with PKCα (10 ng) in 1× phosphorylation buffer in the presence or absence of PMA and GF109204X (GFX). Incorporation of radioactivity was measured, and the transferred membrane was immunoblotted to monitor the input amount of each protein, as indicated.

To determine the PKC-dependent (a and b) and -independent (c) phosphorylation sites of mSK1a, we extracted ^32^P-labeled phosphopeptides (Fig 4A). Purified phosphopeptides were divided into two fractions; one was subjected to phosphoamino analysis and the other was subjected to ^32^P release assay. Phosphoamino analysis revealed that all three phosphopeptides (a, b, and c) exclusively harbor phosphoserine residues within their sequences (Fig 4B). Moreover, ^32^P release assay after Edman degradation revealed that the phosphoserine residues were located in the first or second position in phosphopeptides a and b, and the fourth position of phosphopeptide c, relative to the N-terminus generated by tryptic digestion (Fig 4C). On the basis of this information, several candidates for each phosphopeptide were selected (Fig 4C). We predicted that two sites (S332, S373) were putative sites for phosphopeptide a and b, and one of the others (S140, S225, S338, S369) was the putative site in phosphopeptide c. To determine the exact phosphorylation sites of mSK1a, we performed alanine mutagenesis at each of these residues. Ala mutation at S373 (S373A) resulted in a marked reduction in the level of mSK1a phosphorylation in response to PMA treatment (Fig 4D). Furthermore, comparison of phosphopeptide maps revealed that the reduction of ^32^P-incorporation into the S373A mutant was due to the disappearance of two PMA-responsive phosphopeptides, a and b (indicated by arrowheads in the WT in Fig 4E). By contrast, mutation of S332, the other candidate, had no effect on the level of ^32^P-incorporation. These data suggest that S373 of mSK1a is the only phosphorylation site by PKCα. Because partial digestion occurs frequently in tandem Arg or Lys residues in the protein sequence [42], the two phosphopeptides arising in response to PMA treatment are likely to be generated from partial tryptic digestion of tandem Arg residues in Asp-phosphoSer-**Arg-Arg**-Gly into two similar peptides, Asp-phosphoSer-**Arg** and Asp-phosphoSer-**Arg-Arg**. The *in vitro* PKC assay revealed that S373 is the only PKCα phosphorylation site in mSK1a. PKCα can phosphorylate WT mSK1a and the S225A mutant, but not the S373A mutant (Fig 4F). These data clearly showed that the S373 residue, located in the C-terminus of mSK1a, is the direct phosphorylation site of PKCα.

In addition, Ala mutation at S225 (S225A) resulted in a marginal reduction in the level of mSK1a phosphorylation in response to PMA treatment (Fig 4D). Furthermore, comparison of phosphopeptide maps revealed that the slight decrease of ^32^P-incorporation into the S225A mutant was due to the disappearance of phosphopeptide c (indicated by arrows in the WT in Fig 4E), confirming that S225 is a basal phosphorylation site that is not responsive to PMA treatment. Furthermore, we examined the relationship between different phosphorylation sites. Phosphorylation at S373 was not affected by mutation at S225 (Fig 4F). Conversely, mutation at S373 had no effect on the phosphorylation level at S225. Thus, phosphorylation of S373 and S225 occur under independent control.

### PMA-induced mSK1a inhibition is abolished by S373A mutation

**Fig 5:**
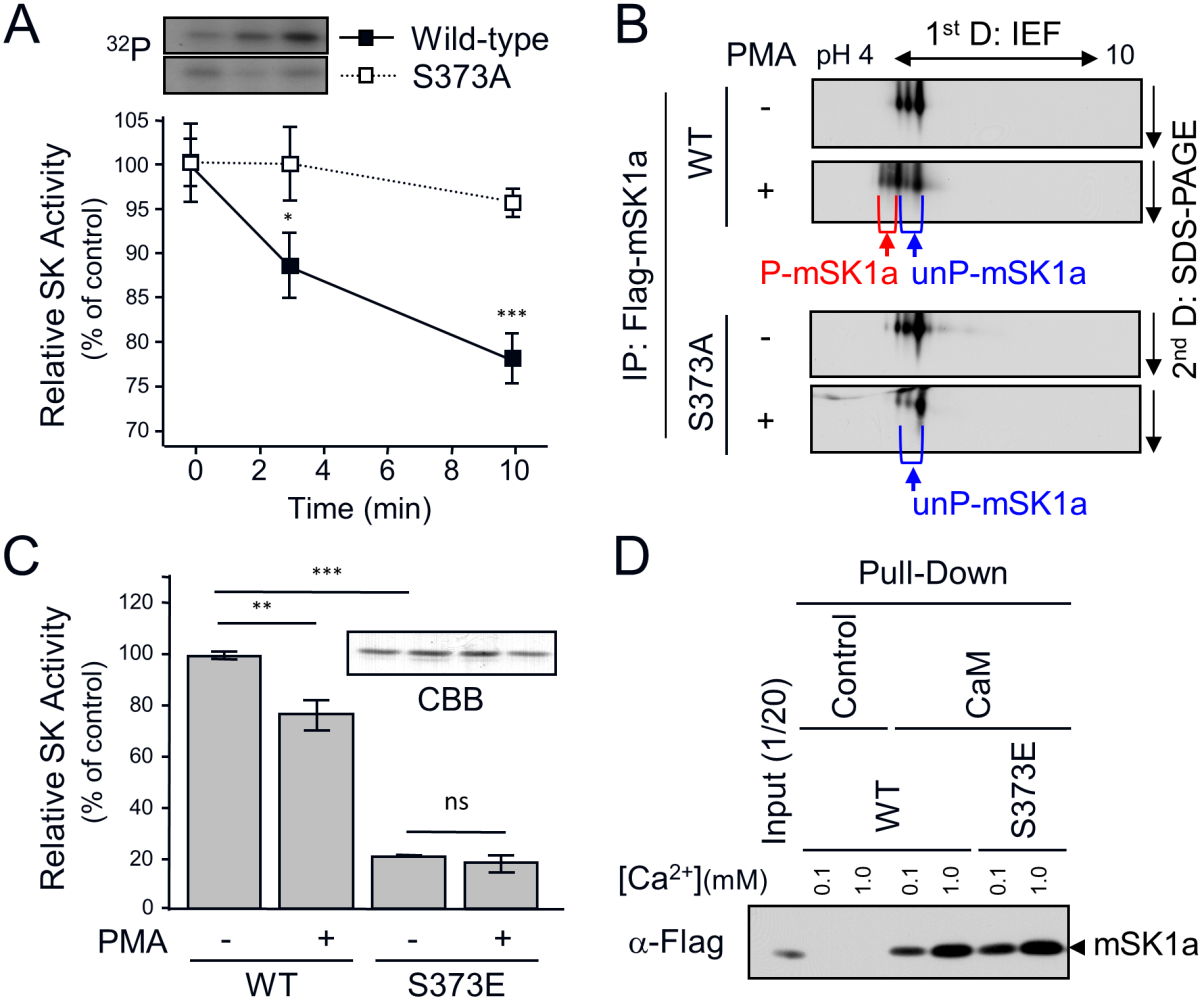
S373 phosphorylation-dependent Inhibition of mSK1a. (A) The effect of mSK1a S373A mutation on PMA-induced inhibition of mSK1a activity. Wild-type (WT) mSK1a or the S373A mutant was transfected into COS-7 cells. One day after transfection, the cells were serum-deprived for 24 hr and treated with 100 nM PMA for the indicated periods of time. Protein extracts from COS-7 cells were assayed for SK activity *in vitro*. SK activity is expressed as the percentage of activity relative to the non-treated control. These data represent the means ± SEM. Inset: time-dependent phosphorylation of WT mSK1a and the S373A mutant (S373A). (B) Approximate stoichiometry of mSK1a phosphorylation. After PMA stimulation, WT mSK1a and its S373A mutant were immunoprecipitated and subjected to 2-D gel electrophoresis. mSK1a was detected using immunoblot analysis using α-FLAG Ab. S373-phosphorylated (P-mSK1a, red) and unphosphorylated (un-P-mSK1a, blue) proteins are indicated below the images. (C) Effect of mSK1a S373E mutation on PMA-induced inhibition of mSK1a activity. Wild-type mSK1a and the S373E mutant were purified from transfected COS-7 cells using α-FLAG Affi-Gel. The same amounts (5 ng) of purified WT mSK1a and S373E mutant were assayed for SK activity. The data are the means ± SEM (t- test, *p < 0.05, **p < 0.01). Inset: The amounts of purified mSK1a and S373E mutant were confirmed by SDS-PAGE followed by Coomassie Blue (CBB) Staining. (D) Calmodulin interaction of WT mSK1a and the S373 mutant. Identical concentrations of mSK1a and the S373E mutant were incubated with either calmodulin-agarose beads or control beads at two different Ca^2+^ concentrations, as indicated. mSK1a bound to beads were assessed by immunoblotting using α-FLAG antibody. See also S1 Fig for additional data about the protein interactions of WT mSK1a and the S373E mutant.

We next examined whether S373 phosphorylation of mSK1a results in an alteration in its activity. Wild-type (WT) mSK1a was phosphorylated and inhibited in a time-dependent manner, whereas the S373A mutant, which was defective in phosphorylation, was refractory to inhibition by PMA treatment over all time periods examined (Fig 5A). Furthermore, we tried to estimate the level of mSK1a phosphorylation by separating phosphorylated and unphosphorylated mSK1a by 2-D gel electrophoresis. In WT transfected cells, but not S373A-transfected cells, PMA treatment shifted mSK1a toward acidic field concomitant with the appearance of additional mSK1a spots, which may be attributable to the generation of a phosphorylation-dependent negative charge in mSK1a (Fig 5B). The phosphorylation level could be roughly estimated at around 20–30% of total mSK1a, roughly correlated with the level of mSK1 inhibition in response to PMA treatment. To determine whether S373 phosphorylation is sufficient to inhibit mSK1a activity, we analyzed the effects of S373E mutagenesis, mimicking the phosphorylated state of S373. The activity of WT mSK1a was inhibited after PMA treatment, whereas the S373E mutant was constitutively inactive irrespective of PMA treatment (Fig 5C), suggesting that S373 phosphorylation plays a significant role in inhibiting mSK1a activity.

Next, we investigated whether alteration in SK activity as a result of the S373E mutation is a result of the aberrant folding of the protein itself. Previous work showed that correctly folded hSK1 protein interacts with Ca^2+^-bound calmodulin [43], which provides an indicator for the proper protein folding of SK1a. Hence, we conducted calmodulin-binding analysis with WT mSK1a and the S373E mutant at two different concentrations of Ca^2+^ ion. We observed that mSK1 interacted with calmodulin in a Ca^2+^ concentration-dependent manner. Importantly, the S373E mutation did not alter its calmodulin interaction under any conditions tested (Fig 5D), suggesting that altered activity of mSK1a S373 mutant is unlikely to aberrant protein folding during protein synthesis. We have further examined whether the S373 mutation of mSK1a influences on protein-protein interactions of mSK1a. While multiple proteins were found co-precipitated with mSK1a, we couldn’t detect any clear difference between WT mSK1a and the S373 mutant (S1 Fig).

## Discussion

SK is regulated by a wide variety of extracellular stimuli in order to mediate multiple physiological functions [1, 3, 5, 44, 45]. However, the detailed molecular mechanisms underlying SK regulation remain incompletely understood. In this study, we explored the phosphorylation-dependent regulation of mSK1a. We observed that S373 of mSK1a is directly phosphorylated by cPKC in response to PMA treatment. Furthermore, this phosphorylation results in the inhibition of mSK1a activity.

Although previous work showed that mSK1a contains several consensus motifs for phosphorylation by protein kinases such as PKA, casein kinase II, and PKC [32], the physiological relevance of putative phosphorylation events in mSK1a have not been rigorously tested. Our results demonstrate that phosphorylation of mSK1a is strongly elicited by PMA, a potent activator of PKC, but not by forskolin, an activator of PKA, indicating that this phosphorylation of mSK1a is kinase-specific. Our results also showed that cPKC including PKCα, phosphorylates mSK1a both *in vivo* and *in vitro*. Mutation at S373 of mSK1a completely abolishes mSK1a phosphorylation in response to PMA treatment, confirming that S373 is the sole site phosphorylated by cPKC. Notably, peptide sequences surrounding S373 residue of mSK1a (-pS-R-R-G-) are consistent with the representative consensus motif for cPKC phosphorylation, -[pS/pT]-[_/F/K/V/L/Q/V]-[K/R]-[K/R/G]- [46], further confirming mSK1a as a novel substrate of activated cPKC within cells. cPKC isoforms including PKCα, -β, and -γ phosphorylate a number of common substrates including GSK-3β, GAP-43, and EGFR [47]. Thus, another cPKC isoform(s) could also be involved in S373 phosphorylation of mSK1a, either alone or together with PKCα, although this was not demonstrated in the present study. Still, the expression and distribution of cPKC isoforms vary between cell types [48]. PKCα are found widely expressed in various cell types whereas others seem to be more restricted in their distributions. Presumably, the relative dominance of PKCα over other cPKC isoforms for the phosphorylation of mSK1a might be influenced by the expression level of each isoform in the given cell type.

**Fig 6:**
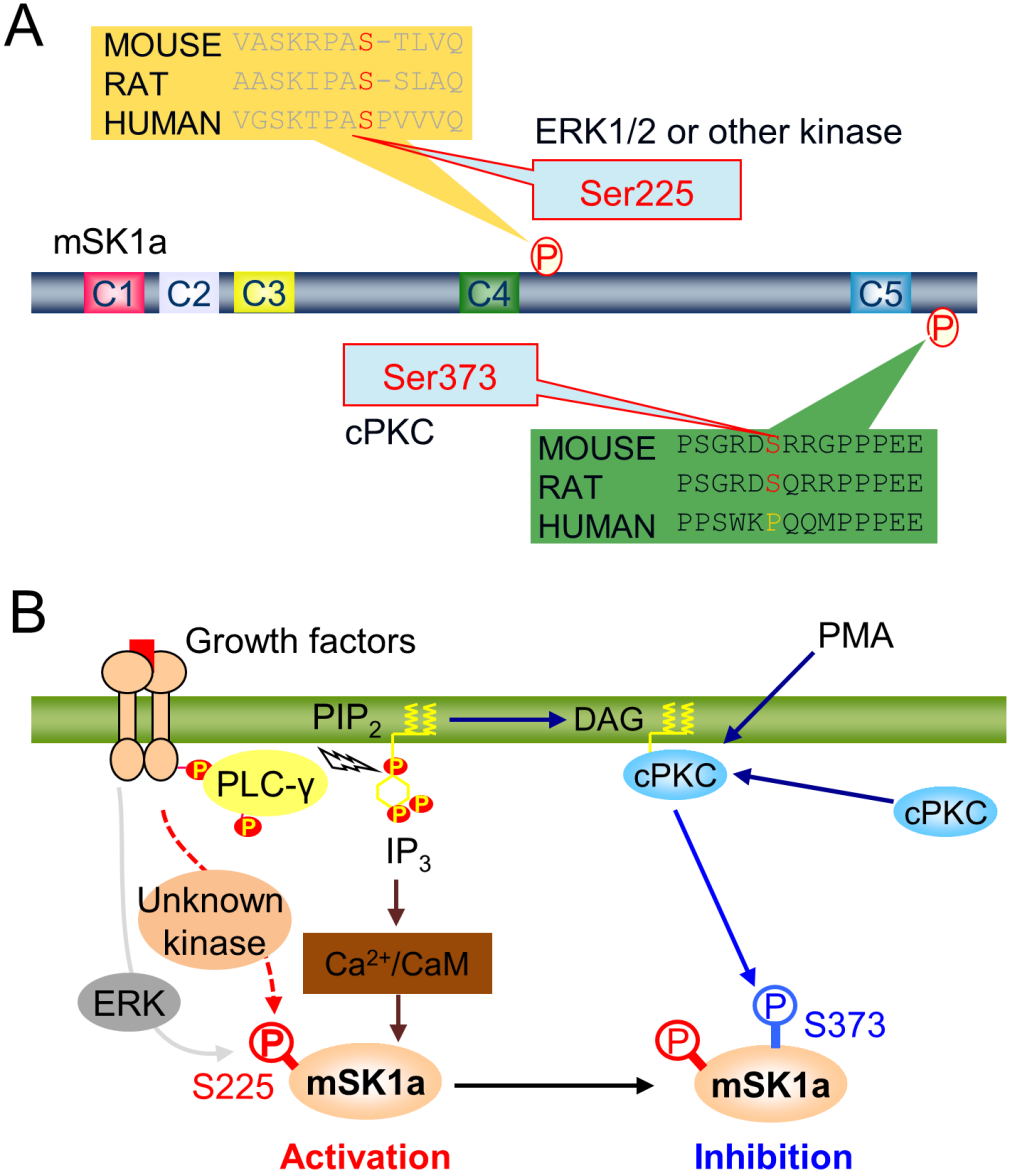
Schematic representation of cPKC-dependent mSK1a regulation. (A) Phosphorylation-dependent regulation of mSK1a. (B) Schematic model for mSK1a regulation in response to extracellular stimuli. Abbreviations: mSK1a (mouse sphingosine kinase 1a), PIP_2_ (phosphatidylinositol 4,5-bisphosphate), DAG (diacylglycerol), cPKC (conventional protein kinase C), PMA (phorbol 12-myristate 13-acetate), ERK (extracellular signal-regulated kinase), CaM (calmodulin), IP_3_, (inositol trisphosphate), PLC-γ (phospholipase C-gamma), P (phosphate group).

The data presented here provide the first direct mechanistic link between cPKC-dependent phosphorylation and mSK1a regulation. Our study revealed that mSK1a is negatively regulated by PKCα-dependent phosphorylation at S373 (Fig 6A). In fact, mSK1a phosphorylation is inversely correlated with mSK1a activity over every time examined, and simple substitution of S373 with Glu is sufficient for the constitutive, dramatic inhibition of mSK1a regardless of PMA treatment. However, it still remains unclear how phosphorylation of mSK1a at this residue modulates enzyme activity. In general, protein modifications such as phosphorylation can change the activity of the modified enzyme through changes in either protein conformation or intermolecular interaction with regulatory binding partner(s). Primarily, the interaction of mSK1a with Ca^2+^-calmodulin complexes was not affected by phosphorylation or mutation at S373, suggesting that the altered activity with phosphorylation or mutation of S373 is unlikely due to interference of the Ca^2+^-calmodulin-dependent regulation of mSK1a. Also, notwithstanding the phosphorylation- or S373E substitution-dependent transition to the inactive form, mSK1a exhibited no visible change in protein–protein interaction(s) (S1 Fig). These observations imply that the reduced kinase activity in the mutants is not the result of disrupted protein–protein interactions with a putative regulatory factor. Therefore, we currently favor a model in which S373 phosphorylation is sufficient to change the intramolecular conformation of mSK1a into an inactive form. Recent study using X-ray crystallography determined the structure of the truncated form of residues 9–364 residue of human SK1 (the full-length contains 384 residues) [49]. To date, however, the structure and function of the proline-rich C-terminus of SK1 is yet to be determined. The results of our current study highlight the potential regulatory importance of the C-terminus of mSK1. In the future, comparison of the protein structures of full-length mSK1a and the S373E mutant may shed light on the molecular mechanisms of regulation, including structural changes resulting from protein phosphorylation at the C-terminus of mSK1a. On the other hand, SK1 protein is exported into the extracellular medium [50, 51], which led us to carefully examine if PMA-induced inhibition of mSK1a is attributable to any unnoticed alteration in the protein level of mSK1a in the tested cells. As shown in multiple figures, neither PMA treatment nor S373 mutation altered the protein level of mSK1a in cells, in contrast to the consistent changes in mSK1a activity. In addition, S1P can be secreted into culture media upon its synthesis. Furthermore, it is noteworthy that the *in vitro* mSK1a activity is consistent with the results from the *in vivo* assay (Fig 2). Collectively, these results support our conclusion that mSK1a is inhibited by S373 phosphorylation, not by the extracellular release of mSK1a protein or of its product S1P. Nonetheless, we cannot exclude the possibility that mSK1a is exported but remains bound to the extracellular surface of the plasma membrane without altering the total mSK1a level in cells. In this regard, it would be interesting to examine if mSK1a S373 phosphorylation can influence the export of mSK1a protein and its extracellular activity on the surface of the plasma membrane in future studies.

We found that mSK1a is phosphorylated in two distinct sites, S225 and S373. Our results identify S373 of mSK1a as the cPKC-dependent phosphorylation site, as well as S225 as the PKC-independent phosphorylation site. A previous report showed that S225 phosphorylation of hSK1a in response to PMA treatment is mediated by ERK1/2, which plays a key role in PMA-induced activation of mSK1a by facilitating both membrane translocation and enzymatic activation of hSK1a [37]. Our result confirmed that phosphorylation at the S225 residue of SK1 is conserved between species. However, it was noteworthy that S225 phosphorylation of mSK1a is barely sensitive to PMA treatment. In fact, sequence alignment between human and mouse mSK1a revealed a meaningful difference in the proline residue immediately adjacent to the phosphorylated S225 residue (Fig 6A). This proline residue is an essential component of the consensus motif for ERK1/2 phosphorylation [46]. Presumably, the absence of P226 residue in mSK1a makes the S225 residue of mSK1a less optimal for the phosphorylation by ERK, which may account for no significant change in S225 phosphorylation by ERK in the cells. In this regard, S225 phosphorylation of mSK1a is unlikely mediated by ERK1/2, but rather by another kinase that is insensitive to PMA treatment, suggesting the significant difference in the signaling pathway to SK1a activation. On the other hand, our current study clearly demonstrated that mSK1a is inhibited through S373 phosphorylation in response to PMA stimulus, which is not consistent with the previous report using hSK1a[37]. Of note, we found that S373 of mSK1a is conserved in mouse and rat, but not in human (Fig 6A), indicating that this phosphorylation and inhibition of mSK1a happens in species-specific manner. Collectively, distinct phosphorylation pattern at two mSK1a phosphorylation sites, S225 and S373 may account for the apparent discrepancy between species regarding PMA-induced SK1a regulation [37]. In particular, our current observation may contribute to better understanding the molecular basis underlying the interspecies differences in sphingolipid metabolism *in vivo*.

In summary, our results demonstrate that mSK1a is inhibited by cPKC-dependent phosphorylation, and that mSK1a phosphorylation is mediated specifically and directly by cPKC. We also demonstrated that phosphorylation at S373, located in the C-terminal regulatory region, plays a pivotal role in the negative regulation of mSK1a. Nonetheless, we have to admit that our study was conduced mostly with cell stimulation with PMA, a type of phorbol esters which does not exist endogenously in mammals, but mimic the action of diacyl glycerol (DAG), potent activator of PKC [52]. In this regards, the physiological relevance of our observation may need to be further validated in the research models of mSK1a-dependent cellular processes including cell proliferation, cell survival, apoptosis, and angiogenesis [2, 45].

In the context of cellular signaling, mSK1a is activated through S225 phosphorylation by unknown kinase probably downstream of growth factor receptor stimulation (Fig 6B). In parallel, phospholipase C (PLC)-dependent cleavage of PIP_2_ generates IP_3_ and diacyl glycerol (DAG), leading to elevation of intracellular Ca^2+^ and cPKC activation. The increase in cellular Ca^2+^ may result in Ca^2+^-calmodulin-dependent activation of mSK1a via direct interaction, whereas cPKC activation may lead to S373 phosphorylation, which results in subsequent inactivation of mSK1a (Fig 6B). This schematic model illustrates the highly dynamic temporal regulation of mSK1a through distinct phosphorylation events triggered by extracellular stimuli.

## Supporting Information

S1 Fig Effect of mSK1a phosphorylation on its molecular interaction. COS-7 cells were transfected with control vector, FLAG-tagged mSK1a wild-type (WT), or its S373E mutant (S373E). After serum deprivation, the cells were treated for 10 min with PMA (100 nM). The cell lysates were incubated with α-FLAG Affi-Gel. The protein complexes co-immunoprecipitated with mSK1a were analyzed with SDS-PAGE followed by Coomassie Brilliant Blue (CBB) staining. Abbreviations: IgGh; Immunoblobulin G heavy chain, IgGl; immunoglobulin G light chain.
(TIF)
