# Supplementary figures and images for: Mouse Sphingosine Kinase 1a Is Negatively Regulated through Conventional PKC-Dependent Phosphorylation at S373 Residue
Source: PLoS One. 2015 Dec 7;10(12):e0143695. doi: 10.1371/journal.pone.0143695 (PMC4671553; doi:10.1371/journal.pone.0143695)

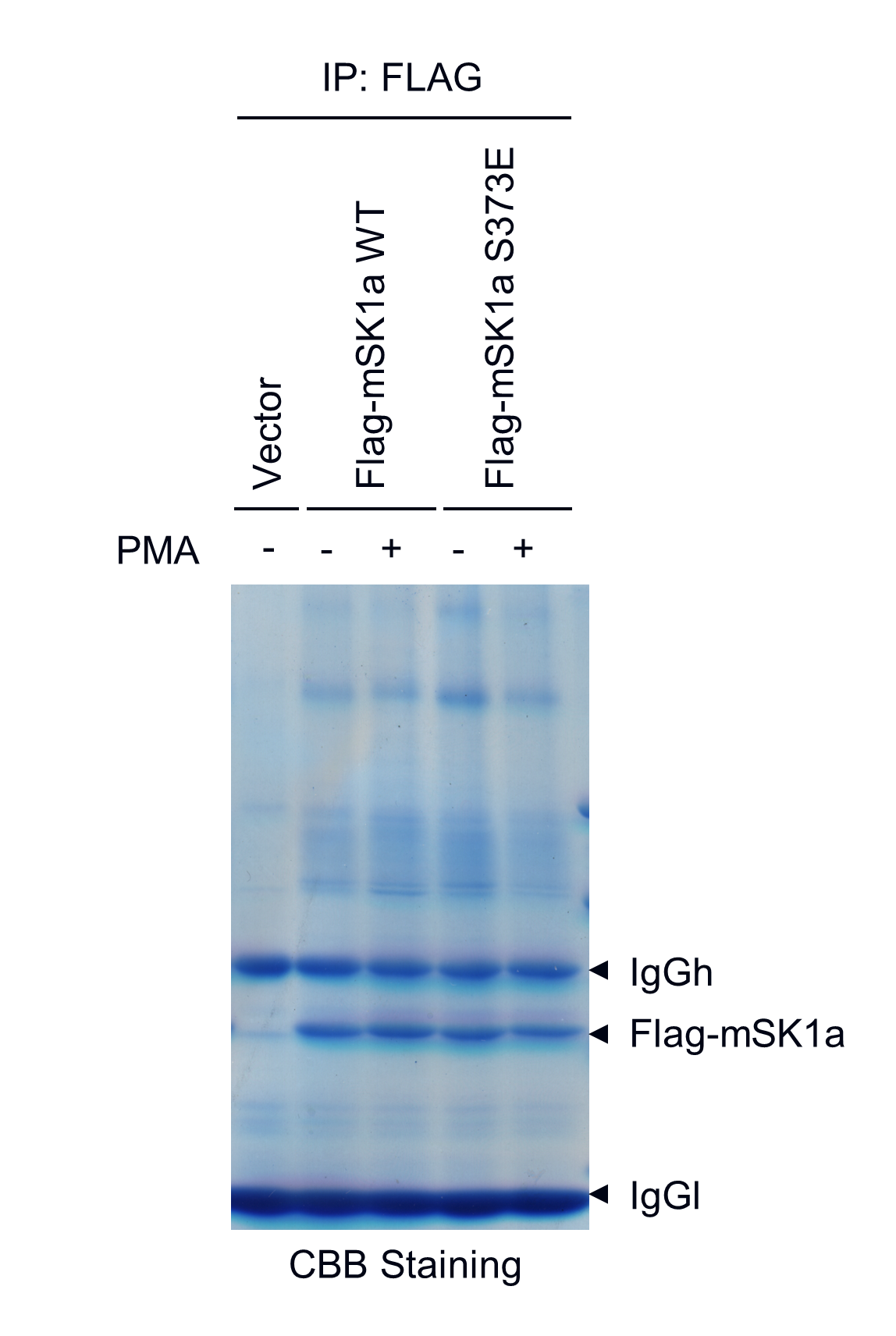

Supplement: S1 Fig — COS-7 cells were transfected with control vector, FLAG-tagged mSK1a wild-type (WT), or its S373E mutant (S373E). After serum deprivation, the cells were treated for 10 min with PMA (100 nM). The cell lysates were incubated with α-FLAG Affi-Gel. The protein complexes co-immunoprecipitated with mSK1a were analyzed with SDS-PAGE followed by Coomassie Brilliant Blue (CBB) staining. Abbreviations: IgGh; Immunoblobulin G heavy chain, IgGl; immunoglobulin G light chain. (TIF) [file pone.0143695.s001.tif]
